# Antibiotic-Induced Manic Episodes: Clinical Recognition, Mechanistic Pathways, and Management

**DOI:** 10.7759/cureus.109861

**Published:** 2026-05-29

**Authors:** Enoch Chi Ngai Lim, Chi Eung Danforn Lim

**Affiliations:** 1 Translational Research Department, Specialist Medical Services Group, Earlwood, AUS; 2 National Institute of Complementary Medicine (NICM) Health Research Institute, Western Sydney University, Westmead, AUS; 3 Data Science Institute, University of Technology Sydney, Sydney, AUS

**Keywords:** antibiomania, antibiotic-associated mania, antibiotic-induced manic episodes, antimicrobial neuropsychiatric toxicity, gut-brain axis

## Abstract

Antibiotic-induced manic episodes describe the emergence of manic or hypomanic symptoms during or shortly after antimicrobial therapy. Although uncommon, these reactions are clinically important because they may be misattributed to primary bipolar disorder, delirium, or neuropsychiatric effects of infection.

Hence, the objective of this study was to synthesize the evidence on antibiotic-induced manic episodes, with emphasis on clinical recognition, plausible mechanisms, management, and verified sequential citations.

A targeted narrative review and reference-verification process was conducted using PubMed/MEDLINE, Google Scholar, publisher webpages, Crossref/DOI records when available, and regulatory safety communications. Searches and source checks were updated through March 2026. Findings were synthesized narratively because the evidence base is mainly case reports, pharmacovigilance data, and reviews.

The literature is dominated by case reports, pharmacovigilance analyses, and systematic or narrative reviews. Prospective denominator-based incidence studies remain lacking. Macrolides, particularly clarithromycin, fluoroquinolones, and antitubercular drugs, account for many published cases. Doxycycline and beta-lactam combinations are also represented in recent reports. Onset is commonly rapid, often within days, and may include insomnia, pressured speech, increased activity, irritability or euphoria, grandiosity, and psychotic symptoms. Most cases improve after the discontinuation of the suspected antibiotic. Severe presentations may require short-term antipsychotic or benzodiazepine treatment. Proposed mechanisms include reduced GABAergic inhibition, monoaminergic effects, including monoamine oxidase-related pathways, CYP3A4-mediated drug interactions, mitochondrial and oxidative stress pathways, and gut-brain axis disruption.

Antibiotic-induced manic episodes should be considered when new manic or psychotic symptoms arise during, or soon after, antibiotic therapy. Management requires prompt medication review, discontinuation or substitution of the suspected agent when clinically feasible, short-term symptomatic psychiatric treatment when indicated, and pharmacovigilance reporting.

## Introduction and background

Mania is defined in the Diagnostic and Statistical Manual of Mental Disorders, Fifth Edition (DSM-5), as a syndrome of abnormally elevated, expansive, or irritable mood with increased energy or activity, accompanied by symptoms such as reduced need for sleep, pressured speech, grandiosity, distractibility, increased goal-directed activity, and impaired judgment. A manic episode typically lasts at least one week, or any duration if hospitalization is required, and causes marked impairment, psychotic features, or clinically significant risk. Hypomania has a similar symptom profile but is shorter and less severe, without psychosis or marked functional impairment [1].

Mania occurring in the setting of physical illness or drug exposure has long been conceptualized as secondary mania. This framework remains useful when a presentation is not fully explained by primary bipolar disorder [2]. Antimicrobial agents are relevant because several antibiotic classes are associated with central nervous system adverse effects, including agitation, insomnia, confusion, hallucinations, psychosis, and seizures [3]. In this review, antibiotic-induced manic episodes refer to manic or hypomanic symptoms that emerge in close temporal relationship to antimicrobial exposure and are not better explained by delirium, primary bipolar disorder, substance use, or the infection itself.

The term "antibiomania" entered wider clinical use in 2002, when Abouesh and colleagues reviewed 21 published cases and 82 World Health Organization spontaneous reports of antimicrobial-associated mania. Clarithromycin, ciprofloxacin, and ofloxacin were among the agents most frequently implicated [4]. Because the term has not been consistently adopted and may appear informal, this review uses the more descriptive term "antibiotic-induced manic episodes", while acknowledging the historical term where necessary for literature retrieval.

Subsequent reviews reinforced the clinical signal while highlighting persistent uncertainty regarding prevalence, mechanisms, and individual susceptibility. A systematic review identified 47 published cases involving 12 antibacterial agents, particularly antitubercular drugs, macrolides, and quinolones [5-7].

Causal attribution remains difficult because infection may independently contribute to neuropsychiatric symptoms through inflammation, fever, metabolic disturbance, sleep disruption, hypoxia, or hospitalization. In one study, individuals admitted with acute mania had higher recent exposure to antimicrobial medications than controls, suggesting that infection, antibiotic exposure, or both may contribute to the clinical presentation [8]. A careful medication timeline, therefore, remains central to evaluation.

Although Parker and Russo recently published an update on antibiotic-associated mania in 2025 [7], the present review differs in several respects. First, it integrates newer mechanistic discussion across GABAergic, monoaminergic, pharmacokinetic, mitochondrial, oxidative stress, and microbiota-gut-brain pathways. Second, it incorporates recent pharmacovigilance evidence and contemporary case reports, including prolonged neuropsychiatric presentations associated with doxycycline exposure [9]. Third, it places greater emphasis on practical clinical recognition, differential diagnosis, and management for general medical and psychiatric practice. Finally, references and DOI records were independently checked to improve bibliographic reliability.

Methods

This article is a narrative review of antibiotic-associated manic and hypomanic episodes. The literature was identified through targeted searches of PubMed/MEDLINE, Google Scholar, publisher webpages, and regulatory safety communications up to March 2026. Priority was given to peer-reviewed case reports, reviews, pharmacovigilance studies, and mechanistic literature relevant to antimicrobial-associated neuropsychiatric toxicity.

Search terms included combinations of “antibiomania”, “antibiotic-induced mania”, “antimicrobial-associated mania”, “clarithromycin mania”, “fluoroquinolone psychiatric adverse effects”, and “antibiotic neuropsychiatric toxicity”. References from retrieved articles were also screened manually for additional relevant reports. Articles were selected based on clinical relevance and discussion of manic or hypomanic presentations temporally associated with antimicrobial exposure. Because this was a narrative review rather than a formal systematic review or meta-analysis, formal risk-of-bias assessment and duplicate independent screening were not performed. Reference details and DOI records were manually checked against primary bibliographic databases and journal webpages before inclusion. Findings were synthesized narratively because the available evidence is heterogeneous and consists predominantly of case reports, spontaneous-report data, pharmacovigilance analyses, and observational evidence.

## Review

Clinical evidence and presentation

Available evidence does not permit a precise denominator-based incidence estimate. Instead, the literature provides a range of reported evidence, from case reports and small systematic reviews to much larger spontaneous-report and pharmacovigilance datasets. These sources are useful for signal detection but are limited by underrecognition, reporting bias, incomplete denominators, and confounding by indication. Table 1 summarizes the main numerical estimates relevant to the apparent frequency and scope of antibiotic-associated manic or psychiatric adverse events.

**Table 1:** Summary of reported numerical evidence relevant to incidence estimation FAERS: FDA Adverse Event Reporting System

Source/evidence type	Numerical finding	What the number can and cannot show
Abouesh et al. 2002: published cases and WHO spontaneous reports [4]	21 published cases and 82 World Health Organization spontaneous reports of antimicrobial-associated mania	Supports an early safety signal, but cannot estimate incidence because the denominator of exposed patients is unknown
Lambrichts et al. 2017: systematic review of antibiotics and mania [5]	47 published cases involving 12 antibacterial agents	Shows the published case range and implicated classes but remains vulnerable to publication and recognition bias
Xie et al. 2024: FAERS fluoroquinolone psychiatric adverse-reaction analysis [10]	27,816 psychiatric adverse drug-reaction reports among 84,777 fluoroquinolone-associated reports	Supports a large pharmacovigilance signal for psychiatric events, but spontaneous reports cannot establish causality or risk per prescription

Across reports, the clinical pattern is recognizable even though causality is often probabilistic. Onset is commonly abrupt after antibiotic initiation, many patients have no previous bipolar diagnosis, dechallenge is frequently followed by improvement, and selected reports describe recurrence after rechallenge [4-6,11]. Reported symptoms include decreased need for sleep, elevated or irritable mood, pressured speech, increased motor or goal-directed activity, distractibility, disinhibition, grandiosity, and impaired judgment. Psychotic symptoms, including hallucinations and persecutory or religious delusions, appear in more severe cases, particularly in reports involving clarithromycin [6].

Macrolides, and clarithromycin in particular, remain the best documented antibiotic group. The early spontaneous-report review, the 2017 systematic review, and the later clarithromycin-focused review all identified clarithromycin as a prominent agent [4-6]. In the case described by Meszaros and colleagues, manic symptoms emerged during treatment for pneumonia, improved after clarithromycin cessation, and later recurred after amoxicillin-clavulanate was reintroduced. This illustrates both the importance of dechallenge and the clinical relevance of recurrence after exposure to a different antibiotic class [11].

Fluoroquinolones warrant particular attention because pharmacovigilance analyses and regulatory guidance recognize clinically relevant neuropsychiatric toxicity. A 2024 FDA (Food and Drug Administration) Adverse Event Reporting System (FAERS) analysis identified mood disorder, psychosis-related, and delirium categories among fluoroquinolone psychiatric adverse-event reports [10]. In 2018, the US FDA strengthened label warnings for systemic fluoroquinolones, including psychiatric adverse reactions, and advised immediate discontinuation when central nervous system adverse effects occur, with substitution by a non-fluoroquinolone antibiotic when possible [12]. These data cannot establish individual causality, but they support a meaningful safety signal.

Tetracyclines have been less prominent than macrolides or fluoroquinolones in earlier literature, but recent reports indicate that they should remain within the differential diagnosis. Lim and colleagues reported a case of doxycycline-associated mania and psychosis after three days of exposure, with neuropsychiatric symptoms persisting for six weeks after discontinuation [9]. This prolonged course differs from the rapid recovery described in many clarithromycin cases, and supports continued monitoring when symptoms are severe or persistent.

Antitubercular agents and beta-lactam combinations also require attention, although their clinical context often complicates attribution. Isoniazid and cycloserine have been represented in systematic reviews of antibiotic-associated mania [4,5], and isoniazid is clinically important because prolonged tuberculosis therapy may limit options for immediate discontinuation. Beta-lactam combinations are less commonly reported, but recurrence after re-exposure to amoxicillin-clavulanate has been documented [11]. In these settings, a drug-by-drug timeline and specialist input are particularly important.

Reliable predictors of risk have not been established. Published cases include children, adults, and older adults; patients with and without psychiatric histories; and both short and prolonged antibiotic courses [4-7,9]. Clinically plausible vulnerability factors include renal or hepatic impairment, older age, pre-existing central nervous system disease, acute sleep disruption, high dose or elevated serum exposure, blood-brain barrier disruption during systemic infection, and co-prescribed drugs that lower seizure threshold or interact pharmacokinetically. These factors should guide monitoring, but their absence does not exclude antibiotic-induced mania.

Clinical context also influences recognition. In emergency settings, agitation may be attributed to infection or delirium. In outpatient settings, emerging hypomania may not be linked immediately to an antimicrobial, especially when agents such as doxycycline or minocycline are prescribed for dermatologic, respiratory, or prophylactic indications. A structured medication timeline is therefore essential. Table 2 summarizes the main antimicrobial classes associated with manic or hypomanic presentations.

**Table 2:** Clinical patterns of antibiotic-associated manic episodes by antimicrobial class CNS: central nervous system; CYP3A4: cytochrome P450 3A4; FAERS: FDA Adverse Event Reporting System

Class/representative agents	Evidence base	Usual timing and presentation	Practical clinical note
Macrolides: clarithromycin; less commonly erythromycin or azithromycin	Early spontaneous-report data, the 2017 systematic review, and a 2026 clarithromycin-focused review identify clarithromycin as the most frequently reported agent [4-6].	Often begins within 1-5 days, with insomnia, pressured speech, agitation, mood elevation or irritability, and psychotic symptoms [6].	Discontinue or substitute when clinically feasible; assess CYP3A4-mediated interactions and concurrent CNS-active drugs.
Fluoroquinolones: ciprofloxacin, ofloxacin, levofloxacin, moxifloxacin	Prominent in earlier reviews and supported by regulatory warnings and FAERS psychiatric adverse-reaction data [4,5,10,12].	Usually rapid in onset; reported features include anxiety, insomnia, agitation, psychosis, delirium, and manic symptoms.	Avoid unnecessary exposure when alternatives exist; discontinue promptly if serious CNS symptoms occur.
Antitubercular agents: isoniazid, cycloserine	Frequently represented in reviews of antibiotics and mania; isoniazid has also been discussed in relation to secondary mania and monoaminergic mechanisms [4,5,13].	May occur later during prolonged therapy; psychosis, mood elevation, agitation, and sleep disruption are reported.	Consider pyridoxine status with isoniazid and seek specialist input because ongoing antimicrobial therapy may be essential.
Beta-lactam combinations and other agents: amoxicillin-clavulanate, cephalosporins	Less common but documented in case literature, including recurrence after amoxicillin-clavulanate re-exposure [11].	Timing is variable and may occur during combination therapy, which can complicate attribution.	Construct a drug-by-drug timeline and consider sequential dechallenge when antimicrobial coverage must continue.
Tetracyclines: doxycycline, minocycline	Rare relative to macrolides and quinolones, but contemporary doxycycline case evidence is available [9].	Onset may occur within days; persistence after discontinuation has been reported [9].	Continue monitoring when symptoms persist beyond expected drug clearance; avoid premature attribution to primary bipolar disorder.
Nitroimidazoles: metronidazole	Neuropsychiatric adverse effects are described in broader antimicrobial reviews, although manic presentations are less common than with macrolides or quinolones [3,7].	Timing is variable; confusion, psychosis, or encephalopathic features may coexist.	Evaluate for delirium, cerebellar toxicity, hepatic dysfunction, and interacting medications.

Proposed mechanisms

No single mechanism accounts for all reported cases. The most consistent biological theme is reduced inhibitory neurotransmission with increased neuronal excitability. Experimental work has shown that clarithromycin can increase excitability in CA3 pyramidal neurons through reduced GABAergic signaling [14]. Fluoroquinolones have also been associated with antagonism of GABA-A receptor activity and increased excitatory potential in in vitro models [15,16]. These mechanisms are consistent with the clinical overlap among antibiotic-associated mania, psychosis, agitation, insomnia, and seizures.

Monoaminergic mechanisms may contribute in selected cases, particularly with antitubercular therapy. Isoniazid has historical and mechanistic relevance because its neuropsychiatric adverse effects helped inform the development of monoamine oxidase inhibitor antidepressants, and isoniazid-associated mania has been described in case-based literature [13]. A monoamine oxidase-related mechanism could increase synaptic monoamines and thereby contribute to mood elevation, insomnia, psychosis, or behavioural activation. Direct patient-level evidence remains limited, so this pathway should be viewed as plausible rather than definitive.

Beyond direct neuronal and monoaminergic effects, pharmacokinetic interactions may also contribute to antibiotic-associated manic symptoms, particularly with macrolides. Clarithromycin and erythromycin are clinically important CYP3A4 inhibitors [17]. Inhibition of this pathway may increase exposure to co-administered neuroactive drugs, including selected antipsychotics, benzodiazepines, calcium-channel blockers, corticosteroids, or other agents, depending on the medication list. This mechanism does not explain cases without interacting drugs, but it is highly relevant to prescribing and risk assessment.

Mitochondrial and oxidative stress pathways may provide another link between antimicrobial exposure and neuropsychiatric activation. In experimental systems, bactericidal antibiotics, including quinolone-class agents, can induce mitochondrial dysfunction and oxidative damage in mammalian cells [18]. Mitochondrial dysfunction has also been implicated more broadly in psychiatric disorders, including mood disorders [19]. Direct evidence linking mitochondrial injury to individual antibiotic-induced manic episodes remains limited, but the pathway is biologically plausible, particularly in severe or prolonged presentations. At present, these pathways should be regarded as hypothesis-generating rather than established causal mechanisms in human antibiotic-induced manic episodes.

A further hypothesis involves disruption of the gut microbiota and the gut-brain axis. Antibiotics can rapidly alter the composition and diversity of the gut microbiome [20,21]. The microbiota-gut-brain axis includes immune signaling, tryptophan metabolism, vagal pathways, the enteric nervous system, and microbial metabolites that may influence central nervous system function [22]. Current evidence does not prove that dysbiosis causes antibiotic-induced mania, although it provides a coherent framework for class-spanning susceptibility and for cases without obvious pharmacokinetic interactions.

These mechanisms are likely to overlap rather than operate in isolation. For example, an older adult receiving clarithromycin for pneumonia may experience infection-related sleep disruption and inflammation, increased exposure to a CYP3A4 substrate, and direct antibiotic-associated neuronal excitability. Similar clinical presentations may therefore arise from different combinations of host vulnerability, infection biology, and antimicrobial pharmacology. Table 3 links the principal mechanisms with their clinical implications.

**Table 3:** Proposed mechanisms and clinical implications CNS: central nervous system; CYP3A4: cytochrome P450 3A4; GABA: gamma-aminobutyric acid

Mechanism	Biological basis and evidence	Most relevant agents	Clinical implications
GABAergic disinhibition and neuronal hyperexcitability	Reduced inhibitory signaling may contribute to insomnia, agitation, psychosis, seizures, and manic activation [14-16]. Evidence is mechanistically plausible, although direct patient-level confirmation remains limited.	Clarithromycin; fluoroquinolones; possibly interacting combinations.	Assess seizure risk, renal function, dose, and co-prescribed pro-convulsant or CNS-active medications.
Monoaminergic and monoamine oxidase-related effects	Isoniazid has historical relevance to monoamine oxidase inhibitor antidepressants, and isoniazid-associated mania has been described in case-based literature [13].	Antitubercular therapy, particularly isoniazid.	Consider monoaminergic activation, insomnia, psychosis, and drug interaction risk when psychiatric symptoms emerge during antitubercular therapy.
CYP3A4-mediated interaction	Macrolide inhibition of CYP3A4 can increase exposure to susceptible co-medications [17]. Evidence is strongest when interacting drugs are present.	Clarithromycin and erythromycin; less relevant to azithromycin.	Review the complete medication list before prescribing and again if symptoms emerge.
Mitochondrial and oxidative stress	Antibiotic-induced mitochondrial dysfunction and oxidative injury may alter neuronal energy metabolism [18,19]. This pathway is biologically plausible but not established for individual clinical cases.	Fluoroquinolones are most often discussed; tetracyclines remain speculative in this context.	Consider prolonged observation when symptoms persist after drug discontinuation.
Gut-brain axis disruption	Antibiotic-induced dysbiosis may alter immune, vagal, endocrine, and metabolite signaling to the brain [20-22]. Direct causality in antibiotic-induced mania remains unproven.	Broad-spectrum agents across classes.	Supports prospective studies incorporating microbiome and inflammatory measures.
Infection and inflammatory confounding	Infection may independently contribute to mania-like or psychotic states, and antibiotic exposure may also mark infection severity [8].	All antibiotic classes.	Evaluate infection severity, delirium, fever, hypoxia, metabolic disturbance, and inflammatory markers.

Diagnosis and management

Recognition begins with a chronological medication and symptom timeline. Clinicians should document the date and dose of antibiotic initiation, symptom onset, sleep change, infection severity, fever, hypoxia, metabolic abnormalities, renal or hepatic impairment, substance exposure, corticosteroid use, and potentially interacting medications. The differential diagnosis should include delirium, encephalitis, sepsis-associated encephalopathy, substance-induced states, corticosteroid-induced mania, withdrawal syndromes, and primary bipolar disorder. Structured adverse-drug-reaction tools, such as the Naranjo algorithm, may support assessment but should not replace clinical judgement [23].

The initial management step is discontinuation of the suspected antibiotic when this can be done safely. If antimicrobial treatment remains necessary, substitution with an agent from a different class should be discussed with infectious disease or antimicrobial stewardship specialists. Fluoroquinolone safety guidance specifically supports immediate discontinuation and switching when significant central nervous system or psychiatric adverse reactions occur [12]. Mild hypomanic symptoms may resolve with cessation and close observation. Severe insomnia, agitation, psychosis, or unsafe behavior may require short-term benzodiazepine and/or antipsychotic treatment while the suspected causative agent clears and medical contributors are addressed [4-7,11]. Mood stabilizers may be considered when symptoms persist beyond expected drug clearance, when episodes recur independently of antibiotic exposure, or when primary bipolar disorder cannot be excluded.

Rechallenge is generally not recommended because recurrence has been documented, and safer alternatives are often available [6,11]. The adverse reaction should be recorded clearly in the medical record, communicated to the patient and primary prescriber, and reported to local pharmacovigilance systems. For patients with established bipolar disorder, antibiotic exposure should be considered when assessing relapse triggers. Sleep, activation, and psychotic symptoms should be monitored proactively during higher-risk antimicrobial courses.

Antibiotic-induced manic episodes are best managed through collaboration between psychiatry, the prescribing team, pharmacy, and, when needed, infectious diseases specialists. Psychiatry can assist with risk assessment, behavioral safety, and symptom-directed pharmacotherapy. The prescribing team can determine whether antimicrobial coverage remains necessary. The pharmacy can review pharmacokinetic interactions, renal dosing, and prior adverse drug reaction history. This shared approach helps avoid unnecessary continuation of an offending agent while also reducing the risk of stopping essential antimicrobial therapy without an appropriate substitute.

Clinical implications and future directions

The evidence base for antibiotic-induced manic episodes is clinically persuasive but methodologically limited. The strongest support comes from temporality, improvement after drug discontinuation, recurrence after rechallenge in selected cases, and convergence across independent case reports and pharmacovigilance systems. The principal limitation is the lack of denominator-based incidence estimates. Spontaneous reporting cannot determine risk per prescription, compare absolute risk across antibiotic classes, or reliably separate antibiotic effects from infection-related effects.

A practical interpretation is that antibiotic-induced mania is rare but clinically credible. Missed recognition may lead to unnecessary psychiatric labeling, avoidable hospitalization, continued exposure to the suspected agent, and delayed antimicrobial substitution. At the same time, over-attribution to antibiotics may obscure primary bipolar disorder, delirium, or severe medical illness. The most defensible approach is probabilistic: assess temporality, evaluate competing causes, observe dechallenge when clinically possible, avoid rechallenge, and document uncertainty when causality remains incomplete.

The recent literature broadens the focus beyond earlier clarithromycin-centered reports. Clarithromycin remains the best documented agent, while fluoroquinolones remain important because regulatory warnings and pharmacovigilance data support a broader neuropsychiatric safety signal. The doxycycline case literature shows that less commonly implicated agents may still be associated with prolonged psychiatric morbidity [9]. This is clinically relevant because doxycycline is widely prescribed in outpatient settings where psychiatric monitoring may be limited. Patient counseling should therefore include advice to report abrupt insomnia, agitation, hallucinations, or marked mood elevation during antibiotic therapy.

Future research should move beyond isolated case reports. Priorities include prospective pharmacovigilance cohorts, electronic health-record studies with prescription denominators, standardized mania-rating measures, and systematic assessment of infection severity. Mechanistic studies should evaluate pharmacogenomic susceptibility, drug-drug interactions, blood-brain barrier vulnerability, inflammatory markers, sleep disruption, mitochondrial markers, monoaminergic pathways, and microbiome changes. Such work is particularly important for commonly prescribed agents such as clarithromycin, ciprofloxacin, levofloxacin, and doxycycline.

## Conclusions

Antibiotic-induced manic episodes are underrecognized adverse drug reactions characterized by new manic or hypomanic symptoms after antimicrobial exposure, often with rapid onset. Macrolides, fluoroquinolones, and antitubercular agents remain the best documented classes, while doxycycline and beta-lactam combinations illustrate the broader clinical spectrum. The management should include early medication review, discontinuation, or substitution of the suspected antibiotic when feasible, short-term symptomatic psychiatric treatment when necessary, careful exclusion of infection-related delirium and primary bipolar disorder, and pharmacovigilance reporting. Clinicians should maintain a high index of suspicion when new mania, psychosis, or severe activation develops during antibiotic therapy.
